# Molecular detection of haemophilic pathogens reveals evidence of *Candidatus Mycoplasma haemobos* in dogs and parasitic ticks in central China

**DOI:** 10.1186/s12917-022-03361-x

**Published:** 2022-07-01

**Authors:** Hongfei Shi, Bozhen Li, Jie Li, Shiwei Chen, Lulu Wang, Zhenzhen Bai, Li Zhu, Baolong Yan, Lunguang Yao

**Affiliations:** 1 Henan Provincial Engineering and Technology Center of Animal Disease Diagnosis and Integrated Control, Henan Provincial Engineering Laboratory of Insects Bio-reactor, Nanyang Normal University, Nanyang, PR China; 2 Department of Parasitology, School of Basic Medical Sciences, Wenzhou Medical University, Wenzhou, PR China

**Keywords:** *‘Candidatus Mycoplasma haemobos’*, Transmission, *Haemaphysalis longicornis*, *Rhipicephalus* (*Boophilus) microplus*, Dog

## Abstract

**Background:**

In addition to *Mycoplasma haemocanis* and *Candidatus Mycoplasma haematoparvum*, a few hemoplasma species that mainly infect other livestock have been detected in dogs. *‘Candidatus Mycoplasma haemobos’* (*Ca. M. haemobos*) has been found in a variety of animals in China. The present study was aimed to investigate the occurrence of *‘Ca. M. haemobos’* infections in dogs and ticks collected from the Henan province, China.

**Results:**

Overall, 55 dog blood samples and 378 ticks on skins were collected from anemic and healthy dogs, and these samples were subjected to PCR, sequence analysis, and identification. The results showed that *Haemaphysalis longicornis* (266) and *Rhipicephalus (Boophilus) microplus* (112) were the only two parasitic ticks on dogs. Molecular detection revealed that 163 *M. haemocanis*, 88 *‘Ca. M. haemobos’* and 32 *Anaplasma platys* positive amplicons could be amplified from dogs, *H. longicornis* and *R. (B.) microplus*. In addition, co-infections (*M. haemocanis* + *A. platys* and *‘Ca. M. haemobos’*+ *A. platys*) could be also detected.

**Conclusions:**

To the best of our knowledge, this is the first molecular evidence of *‘Ca. M. haemobos’* natural infection in dogs and tick species identified as *H. longicornis* and *R. (B.) microplus* from China.

## Background

Hemoplasmas are small unculturable bacteria that reside on the surface of erythrocytes. Based on the sequences of *16S rRNA* analysis, these pathogens were reclassified as genus *Mycoplasma* [1]. *Mycoplasma haemocanis* (*M. haemocanis*) and *Candidatus Mycoplasma haematoparvum* (*C. M. haematoparvum*) are the two main hemoplasmas that infect dogs [2, 3]. However, a few hemoplasma species that mainly infect other livestock have been detected in dogs: in China and Japan *Candidatus Mycoplasma haemominutum*, which mainly infects cats, was found in blood samples collected from dogs [4, 5]. In the USA, *Mycoplasma ovis* mainly infects goats and sheep and was found in splenic hemangiosarcoma samples collected from dogs [6]. In Australia *‘Candidatus Mycoplasma haemobos’* (*‘Ca. M. haemobos’*) was detected in two blood samples collected from dogs [7, 8].

*‘Candidatus Mycoplasma haemobos’* is an emerging pathogen found in a variety of hosts, including cattle (*Bos taurus*) [9–13], water buffalo (*Bubalus bubalis*) [14], red deer (*Cervus elaphus*) [15], fallow deer (*Dama dama*) [15], roe deer (*Capreolus capreolus*) [15], goats (*Capra aegagrus hircus*) [16], and sheep (*Ovis aries*) [16]. These natural infections can cause anemia [13, 16, 17], transient fever [16], lymphadenopathy [13, 17], anorexia [18], weight loss and decreased milk production [18, 19]. Natural infections of *‘Ca. M. haemobos’* have been found in Africa [20, 21], Asia [10, 16, 22, 23], Europe [12, 15] and South America [14, 24]. In backyard farms in central China, *‘Ca. M. haemobos’* has been verified in goats and sheep, and *Rhipicephalus (Boophilus) microplus* (*R. (B.) microplus)* ticks can serve as a vector and reservoir in the transmission of *‘Ca. M. haemobos’* [16, 25]. In these backyard farms, dogs are usually housed for guarding homes and livestock in grasslands, so the dogs share the same living areas with goats and sheep. Considering that the *R. (B.) microplus* can parasitize all three species of animals [26–28], whether the ticks could transmit *‘Ca. M. haemobos’* to dogs is unknown. This study aimed to investigate the occurrence of ‘Ca. M. haemobos’ infections in dogs and ticks collected from the Henan province in central China. In addition, other pathogens including *Ehrlichia canis*, *Anaplasma platys* (*A. platys*) [29], *Babesia* and *Theileria* [30] were also investigated due to similar anemia symptoms in dogs.

## Methods

### Animals, blood and tick sample collection

The sample collection were conducted from April to July during the peak season of *‘Ca. M. haemobos’* infections and tick activities between 2019 and 2020 in rural areas of Henan Province adjacent to Hubei province, central China, where *‘Ca. M. haemobos’* epidemics had been confirmed [16], the landform in the territory is dominated by shallow mountains and hills, while the climate is north subtropical monsoon continental warm and humid climate with abundant rainfall. A total of 55 EDTA-anticoagulated blood and serum samples were collected from the anterior tibial vein of the dogs with infesting ticks, including 35 sick dogs with anemia diagnosed by the vet in the rural veterinary clinic and 20 dogs considered as clinically healthy in the backyard farms. At the moment of blood sample collection, none of the dogs were under antibiotics or acaricide treatment. Complete blood counts of all EDTA-anticoagulated blood samples were made, and the dogs were reclassified as anemic or healthy based on results compared with reference ranges (pack cell volume (PCV): 0.37–0.55 L/L). After determining the complete blood counts of all dog blood samples, six dogs previously considered as clinically healthy were reclassified as anemic, and all 35 sick dogs presenting with anemia were verified by the results. The study then included 41 anemic dogs and 14 healthy dogs based on PCV. The remaining blood samples were stored at −80°C for molecular analysis. In addition, all ticks (378) from the body surfaces of the dogs were collected and treated individually as in previous work [16].

### Tick identification

All ticks were first identified using morphological and taxonomic identification keys and then verified by molecular analysis [16]. The ticks were homogenized in 1 mL of phosphate buffered saline buffer, then each of the composite 200 µL homogenates was used for DNA extraction with the *EasyPure*® Genomic DNA Kit (Transgen Biotech, China). The primers of T1B and T2A as reported by [31] were used to amplify the 12S rRNA gene, and amplicons were purified using the gel extraction kit (Omega, China) and the purified products were directly sequenced in both directions using an ABI automated A373 sequencer (ABI, USA).

### Primer selection, DNA extraction, amplification, and sequencing

For amplifying and analyzing the target gene of potential pathogens for dog blood samples, DNA was extracted using an EasyPure Blood Genomic DNA kit (TransGen Biotech, China) according to manufacturer instructions. DNA samples were used as templates in PCR reactions carried out as previously described [32, 33]; in addition, DNA of a *M. wenyonii* strain and DEPC-treated water were used as a positive control and a negative control, respectively in all PCR reactions. The primer set (5’-ACGAAAGTCTGATGGAGCAATA-3’ and 5’-ACGCCCAATAAATCCG(A/G)ATAAT-3’) designed to detect *Mycoplasma haemofelis* had previously been proved to be effective in amplifying the partial *16S rRNA* gene of *M. haemocanis*, *C. M. haematoparvum*, *Candidatus Mycoplasma haemominutum*, *Mycoplasma ovis*, *Candidatus Mycoplasma haemovis*, *Mycoplasma wenyonii*, and *‘Ca. M. haemobos’* [16, 33]. In addition, Apla-sense and ECB for *Ehrlichia canis* and *Anaplasma platys* (*A. platys*) [29], BTH 18S 1st F/R and BTH 18S 2nd F/R primers for *Babesia* and *Theileria* [30] were also used. After the first molecular screening, all positive amplicons were visualized on an agarose gel following electrophoretic separation and recovered using an EasyPure PCR purification kit (TransGen Biotech, China), then sequenced by an ABI 3100 sequencer (ABI, USA). All sequences were aligned with relevant sequences published in the NCBI databases using a BLAST search. Then, all the *‘Ca. M. haemobos’* positive samples were further subjected to phylogenetic analysis by amplify longer fragments (1393 bp) of *16S rRNA* using primers MHBforw and MHBrev [32]. Similarly, all tick DNAs were also subjected to PCR tests as for blood.

### Phylogenetic analysis

Sequences of the long *16S rRNA* gene amplicons were compared with the CLUSTALW program using the strains from Switzerland (clones 307 and 311), Japan (cattle nos. 18, B2.16 and B2.20), Germany (BovHM-2 and BovHM-7), Brazil (Bov 165), Cuba (C115), Malaysia (I924712) and China (HN1804, HN1807, China, CMboTWN01, CMboTWN02, and CMboTWN01). Phylogenetic analysis was performed using Molecular Evolutionary Genetics Analysis version 6 (MEGA6) [34] based on neighbor-joining criterion and the Kimura 2-parameter model. Stability of the trees was tested by bootstrap analysis using 1,000 replicates.

### Statistical analysis

Statistical analysis for significant differences for the tick infestation levels between healthy dogs and anemic dogs was performed by using SPSS 17 on T test. *P*-value<0.05 was considered as threshold for statistical significance.

## Results

### Ticks

Among the 378 tick samples, 329 ticks were collected from dogs with anemia, and 49 ticks were collected from healthy dogs. Further morphological and taxonomic keys examination identified 89 male ticks and 289 females. These ticks were identified to 2 species of 2 genera of the family Ixodidae: 266 *H. longicornis* (36 and 230 collected from healthy dogs and anemic dogs, respectively) and 112 *R. (B.) microplus* (13 and 99 collected from healthy dogs and anemic dogs, respectively). Significant differences had been found for the tick infestation levels between healthy dogs and anemic dogs (T test, *P*<0.001, α=0.05). The tick species, their host origins, sex and numbers are shown in Table. 1.

**Table 1:** Species of ticks collected from animal hosts in this work

Species	Hosts	No. of ticks		
		Male	Female	Total
*Haemaphysalis longicornis*	Healthy dogs	7	29	36
	Anemic dogs	64	166	230
*Rhipicephalus (Boophilus) microplus*	Healthy dogs	3	10	13
	Anemic dogs	15	84	99
Total		89	289	378

### Pathogens

After tick species identification, all samples were divided into six groups for analysis: group 1 included blood samples collected from healthy dogs; group 2 included blood samples collected from anemic dogs; group 3 included *H. longicornis* samples collected from healthy dogs; group 4 included *H. longicornis* samples collected from anemic dogs; group 5 included *R. (B.) microplus* samples collected from healthy dogs, and group 6 included *R. (B.) microplus* samples collected from anemic dogs. After screening for the presence of the short fragment of *16S rRNA* of hemoplasmas in dog blood samples and tick samples by PCR the percentages of hemoplasmas positive rates in six groups showed in Table 2. Sequencing and aligning in the NCBI databases using a BLAST search indicated the positive amplicons were including 163 *M. haemocanis* and 88 *‘Ca. M. haemobos’* positive samples, and no *C. M. haematoparvum*, *Candidatus Mycoplasma haemominutum*, *Mycoplasma ovis*, *Candidatus Mycoplasma haemovis* and *Mycoplasma wenyonii* was detected in this work. Further screening for other pathogens revealed that 32 samples were positive for *A. platys*, and no *Ehrlichia canis, Babesia* and *Theileria* was detected in all samples. As Table 2 shows, the co-infections (*M. haemocanis* + *A. platys* and *‘Ca. M. haemobos’* + *A. platys*) could be detected in group 2, group 4, and group 6, and no other co-infections had been observed in this work. The frequencies of single infections of *M. haemocanis* in dogs, *H. longicornis* and *R. (B.) microplus* were 89.5% (17/19), 94.4% (117/124) and 95.0% (19/20). Similarly, single infections of *‘Ca. M. haemobos’* in dogs, *H. longicornis* and *R. (B.) microplus* were 81.8% (9/11), 81.8% (18/22) and 94.3% (50/53). More details are given in Table 2.

**Table 2:** Frequency of tick‐borne pathogens in ticks and dogs blood samples

Samples	Number	Hemoplasmas (percentage)	*M. haemocanis* (sum)	*‘Ca. M. haemobos’*(sum)	*A. platys* (sum)	*M. haemocanis* + *A. platys*	*‘Ca. M. haemobos’*+ *A. platys*
Group 1	14	4 (28.6%)	3	1	0	0	0
Group 2	41	26 (63.4%)	16	10	6	2	2
Group 3	36	6 (16.7%)	4	2	1	0	0
Group 4	230	144 (62.6%)	120	22	17	7	4
Group 5	13	4 (30.8%)	2	2	0	0	0
Group 6	99	69 (69.7%)	18	51	8	1	3

*M. haemocanis* Mycoplasma haemocanis, *‘Ca. M. haemobos’* Candidatus Mycoplasma haemobos, *A. platys* Anaplasma platys

### Sequence analysis of *‘Ca. M. haemobos’*

After longer amplicons sequencing five sequence types were observed in these samples as Table 3 showed. Five strains were selected as representative for analysis, HN1804 strain (GenBank Accession number MH388478) and HN1807 strain (GenBank Accession number MH388476) described previously [16]; HN1921 (GenBank Accession number MW463059), HN1933 (GenBank Accession number MW463060) and HN1948 (GenBank Accession number MW463061) were three new sequence types. As showed in Table 3, In group 1, group 3, and group 5 only new sequence type strains were observed, and in group 2, group 4, and group 6 previous and new sequence type strains were observed. In total, 88 positive samples with the new HN1933 sequence type showed the highest frequency (30/88), and the previous HN1807 sequence type showed the lowest frequency (10/88). Among the three sources of samples, the highest *‘Ca. M. haemobos’* positive rate was observed in *R. (B.) microplus* at 47.32% (53/112) followed by rates in dogs and *H. longicornis* of 20.00% (11/55) and 9.02% (24/266).

**Table 3:** Frequency of *‘Ca. M. haemobos’* sequence types in ticks and dogs blood samples

Sequence type	Dog blood (11/55)		H.L. (24/266)		R.B.M. (53/112)		Sum (88/433)
	Group 1 (1/14)	Group 2 (10/41)	Group 3 (2/36)	Group 4 (22/230)	Group 5 (2/13)	Group 6 (51/99)	
HN1804	0	1	0	3	0	7	11
HN1807	0	0	0	2	0	8	10
HN1921	0	2	0	5	0	10	17
HN1933	1	4	2	7	1	15	30
HN1948	0	3	0	5	1	11	20

*H.L.* Haemaphysalis longicornis*, B.M.* Rhipicephalus (Boophilus) microplus

**Fig. 1:**
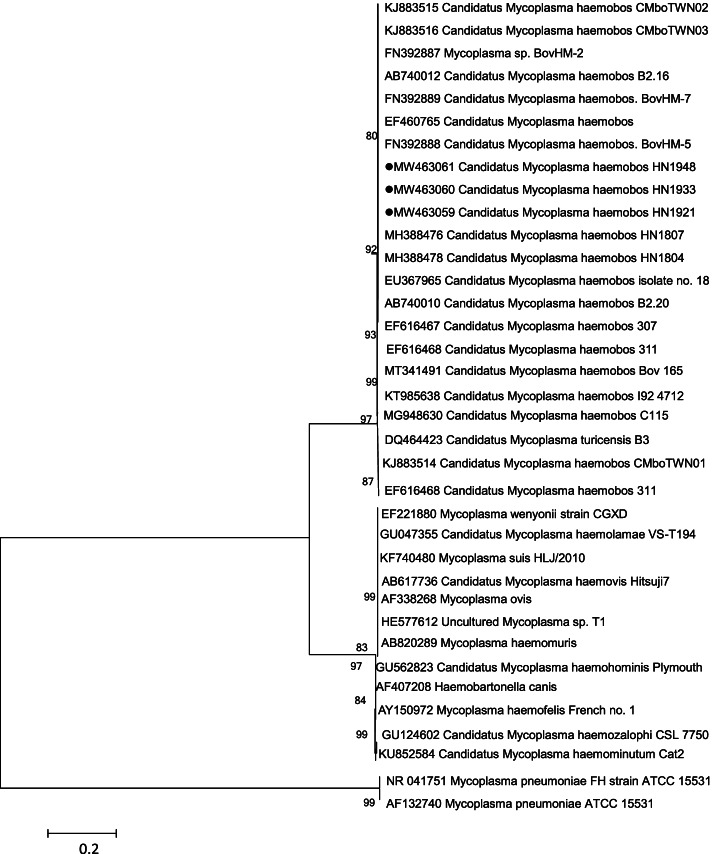
Phylogenetic analysis of *‘Ca. M. haemobos’* from dogs, *Rhipicephalus (Boophilus) microplus* and *Haemaphysalis longicornis* in Central China, and reference strains using the *16S rRNA* sequences. New isolates in this work are highlighted with a symbol (·).

Comparative analysis of the three new representative isolates and other strains in Switzerland (clones 307 and 311), Japan (cattle nos. 18, B2.16 and B2.20) and China (HN1804, HN1807, China, CMboTWN01, CMboTWN02, and CMboTWN01) revealed a nucleotide sequence similarity of 98.6%–99.8%. Using a proposed taxonomic key of *‘Ca. M. haemobos’* in previous research [10, 13, 16, 32], phylogenetic analysis of the *16S rRNA* sequence (Fig. 1) characterized all three representative strains as *‘Ca. M. haemobos’*. In addition, the strains in this work were most closely related to the strain isolated from Central China, and were most distantly related to those from Switzerland (clones 307 and 311) and Japan (cattle nos. 18, B2.16, and B2.20).

## Discussion

*Mycoplasma haemocanis* and *C. M. haematoparvum* are the two main hemoplasmas infections in dogs [2, 3]. About ten years ago, reports indicated that the DNA of heterogenous hemoplasma species, including *Candidatus Mycoplasma haemominutum* and *Mycoplasma ovis*, could be detected in samples collected from dogs [4–6]. In Australia *‘Ca. M. haemobos’* was detected in a healthy dog [8] and then co-infection with *A. platys* was detected in an anemic dog [7]. However, with limited positive samples in previous work, the evidence of a connection between *‘Ca. M. haemobos’* and infections in dogs was still unclear. We studied 14 healthy dogs and 41 anemic dogs, and the results showed that one healthy dog and ten anemic dogs were positive for *‘Ca. M. haemobos’*. Among the positive anemic dogs, eight were solely infected, suggesting that *‘Ca. M. haemobos’* should play a role in the progress of syndrome. Considering that one healthy dog was also positive, further work with experimental infections in dogs would clarify the pathogenesis of *‘Ca. M. haemobos’* in dogs. In China, *‘Ca. M. haemobos’* were first found in cattle in Guangxi Province [35] and were then described in goats and sheep [16]. The present work is the first report about *‘Ca. M. haemobos’* in dogs in China, and phylogenetic analysis showed that all the new strains in the present study were most closely related to isolates from goats and sheep described in previous work. Dogs in this work shared the same habitation with those small ruminates, and previous work had shown that *R. (B.) microplus* can transmit *‘Ca. M. haemobos’* [16, 25]. Whether tick vectors are involved in the transmission *‘Ca. M. haemobos’* to dogs needs to be investigated.

Previous studies showed that *R. (B.) microplus* ticks could serve as a vector and reservoir in the transmission of *‘Ca. M. haemobos’* [16, 25]: *R. (B.) microplus* ticks carrying *‘Ca. M. haemobos’* could be found in naturally infected goats, sheep and grassland and experimental infections showed larval ticks can transmit *‘Ca. M. haemobos’* to BALB/c mice during feeding [16, 25]. Considering the hosts of *R. (B.) microplus* ticks are diverse, it is unclear whether the transmission of *‘Ca. M. haemobos’* could infect other animals. Then the *R. (B.) microplus* ticks associated with these dogs were investigated. We found that the *‘Ca. M. haemobos’* positive ticks could be detected from both positive and negative dogs. Certainly it cannot rule out the possibility that the presence of ‘Ca. M. haemobos’ in ticks collected from PCR-negative dogs could be for the existence of a previous blood meal. In addition, only adult ticks were found on infesting dogs, unsurprisingly one previous work had been showed the adult ticks were main paratized ticks on the dogs in central aand eastern China [36]. However, the conclusion that infections in dogs were caused by these ticks could be not supported because other positive ticks were involved in the animia syndrome. Considering the high positive rate of the *R. (B.) microplus* ticks and dogs, it is necessary to conduct experimental infections to investigate details of the role of transmission of *‘Ca. M. haemobos’* in dogs. In addition, regarding the widespread distribution of *R. (B.) microplus* in China [37, 38], whether other hosts of *R. (B.) microplus* ticks such as rabbits, pigs, horses, and donkeys are susceptible to *‘Ca. M. haemobos’* is unknown, and thus it is urgent to investigate the prevalence of *‘Ca. M. haemobos’* in other animals exposed to *R. (B.) microplus* ticks and evaluate the risk of *‘Ca. M. haemobos’* to livestock.

The transmission mode of *‘Ca. M. haemobos’* is unclear. One study suggested that *Haematobia irritans*, *Stomoxys calcitrans, Tabanus bovines,* and *Tabanus bromius* were potential vectors for spreading *M. wenyonii* and *‘Ca. M. haemobos’* [39]. Another study indicated that *Dermacentor andersoni* could transmit *M. wenyonii* [40, 41] reported that four species of ticks (*D. reticulates*, *Haemaphysalis inermis*, *Ixodes ricinus*, and *D. marginatus*) are unlikely vectors for *M. wenyonii* and *‘Ca. M. haemobos’*. We previously showed that *R. (B.) microplus* ticks could transmit *‘Ca. M. haemobos’* [16, 25]. However, whether other species of tick can carry or transmit *‘Ca. M. haemobos’* is unclear. Southern Henan province is a region with great tick diversity [42], and a large number of tick-borne diseases have been recorded there [43–46]. In the present work 24 *H. longicornis* ticks tested positive for *‘Ca. M. haemobos’*, including two ticks collected from healthy dogs, thus this result indicated that the two ticks either acquire *‘Ca. M. haemobos’* from the previous host, or carry *‘Ca. M. haemobos’* from the previous stage. In either case, there is no doubt that *‘Ca. M. haemobos’* could sustain in *H. longicornis* ticks for a period. In *R. (B.) microplus* ticks, research has verified that *‘Ca. M. haemobos’* can be passed transovarially and negative ticks can acquire *‘Ca. M. haemobos’* in experimentally infected animals [25]. It remains unclear whether *H. longicornis* ticks have similar ability to transmit *‘Ca. M. haemobos’* and further work is needed for clarification.

*Anaplasma platys* and *M. haemocanis* were also detected in dogs, *R. (B.) microplus*, and *Haemaphysalis longicornis. A. platys* were previously detected in domestic animals in ten Provinces of China, including dogs in Henan Province. *R. (B.) microplus* and *H. longicornis* were also found positive to *A. platys* [47]. To date, only one study reported *M. haemocanis* infections in dogs in China [48]. The tested dogs had a history of tick infestation; however, the exact tick species were not recorded. Some studies have suggested that *Rhipicephalus sanguineus* (*R. sanguineus*) should be the potential vector for *M. haemocanis* [49–52]. In the present study, no *R. sanguineus* samples were collected, but *R. (B.) microplus* and *H. longicornis* were detected to be positive to *M. haemocanis*. Engorged *H. longicornis* larvae were not infected with *M. haemocanis* from domestic cats (*Felis catus*), eastern gray squirrels (*Sciurus carolinensis*), marmots (*Marmota monax*), raccoons (*Procyon lotor*), striped skunks (*Mephitis mephitis*), Virginia opossums (*Didelphis virginiana*), or white-tailed deer (*Odocoileus virginianus*) in the USA [53], but in dogs the association of *M. haemocanis* and *H. longicornis* is unclear. This work indicated the potential of vectors in transmission of *M. haemocanis* in dogs, but further research is needed for clarification. We showed, for the first time, that *R. (B.) microplus* could carry *M. haemocanis*. Moreover, *A. platys* could be involved in co-infections (*M. haemocanis* + *A. platys* and *‘Ca. M. haemobos’* + *A. platys*) in anemic dogs, *H. longicornis* and *R. (B.) microplus.* Similarly, a previous study [54] showed about 11.11% positive rate for ticks collected from dogs and sheep in Xinyang city in China near the Nanyang area. In that study, there were co-infections with two pathogens, and *A. platys* co-infection was recorded; however, *M. haemocanis* and *‘Ca. M. haemobos’* detection were not done. These tick species distributions are consistent with those from previous studies [55–57], suggesting that *H. longicornis* and *R. (B.) microplus* were the dominant tick species in central China. Furthermore, the ticks in this area could carry a variety of pathogens and infest multiple livestock species, and thus it is urgent to evaluate the spread risk of new diseases as *‘Ca. M. haemobos’* moves to new hosts via these potential vectors. Meanwhile, co-infection [58] might be a factor affecting the disease process in target hosts and in vector transmission ability. Molecular surveys of haemoplasmas in ticks associated with dogs have been also examined in *Rhipicephalus sanguineus sensu lato* [49, 59], but no co-infection has been documented.

## Conclusions

*‘Ca. M. haemobos’* infections in dogs, single or co-infection were verified by PCR, sequencing, and phylogenetic analysis. We provided molecular evidence for natural infections of *‘Ca. M. haemobos’* in dogs, and information showing that *H. longicornis* can carry *‘Ca. M. haemobos’*.

## Ackowledgements

We thank LetPub (www.letpub.com) for its linguistic assistance during the preparation of this manuscript.

## Data Availability

The datasets generated and analysed during the current study are available in the GenBank repository at [www.ncbi.nlm.nih.gov/nucleotide/MW463059], [www.ncbi.nlm.nih.gov/nucleotide/MW463060] and [www.ncbi.nlm.nih.gov/nucleotide/MW463061].

## Abbreviations

*M. haemocanis*: *Mycoplasma haemocanis*
*C.** M. haematoparvum*: *Candidatus Mycoplasma haematoparvum*
*‘Ca.** M. haemobos’*: *Candidatus Mycoplasma haemobos*
*H.** longicornis*: *Haemaphysalis longicornis*
*R.** (B.) microplus*: *Rhipicephalus (Boophilus) microplus*
*A. platys*: *Anaplasma platys*
*C. M. haemominutum*: *Candidatus Mycoplasma haemominutum*
